# 3,4,5-Trimethoxybenzoate of Catechin, an Anticarcinogenic Semisynthetic Catechin, Modulates the Physical Properties of Anionic Phospholipid Membranes

**DOI:** 10.3390/molecules27092910

**Published:** 2022-05-03

**Authors:** Elisa Aranda, José A. Teruel, Antonio Ortiz, María Dolores Pérez-Cárceles, José N. Rodríguez-López, Francisco J. Aranda

**Affiliations:** 1 Departamento de Bioquímica y Biología Molecular-A, Universidad de Murcia, 30100 Murcia, Spain; elisa.aranda@um.es (E.A.); teruel@um.es (J.A.T.); ortizbq@um.es (A.O.); neptuno@um.es (J.N.R.-L.); 2 Departamento de Medicina Legal y Forense, Facultad de Medicina, Instituto de Investigación Biomédica (IMIB-Arrixaca), Universidad de Murcia, 30120 Murcia, Spain; perezcarcelesmariadolores@gmail.com

**Keywords:** catechin, dimyristoylphosphatidylserine, phospholipid membranes

## Abstract

3,4,5-Trimethoxybenzoate of catechin (TMBC) is a semisynthetic catechin which shows strong antiproliferative activity against malignant melanoma cells. The amphiphilic nature of the molecule suggests that the membrane could be a potential site of action, hence the study of its interaction with lipid bilayers is mandatory in order to gain information on the effect of the catechin on the membrane properties and dynamics. Anionic phospholipids, though being minor components of the membrane, possess singular physical and biochemical properties that make them physiologically essential. Utilizing phosphatidylserine biomimetic membranes, we study the interaction between the catechin and anionic bilayers, bringing together a variety of experimental techniques and molecular dynamics simulation. The experimental data suggest that the molecule is embedded into the phosphatidylserine bilayers, where it perturbs the thermotropic gel to liquid crystalline phase transition. In the gel phase, the catechin promotes the formation of interdigitation, and in the liquid crystalline phase, it decreases the bilayer thickness and increases the hydrogen bonding pattern of the interfacial region of the bilayer. The simulation data agree with the experimental ones and indicate that the molecule is located in the interior of the anionic bilayer as monomer and small clusters reaching the carbonyl region of the phospholipid, where it also disturbs the intermolecular hydrogen bonding between neighboring lipids. Our observations suggest that the catechin incorporates well into phosphatidylserine bilayers, where it produces structural changes that could affect the functioning of the membrane.

## 1. Introduction

There is compelling evidence from biochemical and biological studies that green tea catechins can produce diverse beneficial effects on cancer in humans, including prevention of cancer, synergistic anticancer effect, and inhibition of metastasis [1]. Catechins display many anti-carcinogenic and anti-mutagenic promising protective effects in cancer, including breast, esophagus, prostate, stomach, small intestine, colon, liver, and lung [2]. A number of in vitro, in vivo, and clinical studies have established that catechins produced their anticancer effects by way of the modification of several signaling routes, most of which are provoked by the interaction between catechins and an array of membrane proteins, intracellular molecules, membrane microdomains, and the plasma membrane itself [3].

A series of indications point to the membrane as a potential target for the anticancer role of catechins. Catechins increase the stiffness of different cancer cells through alteration of membrane organizations both in melanoma [4] and lung cancer [5]. The interaction of catechins with phospholipids bilayer membranes affect PKC activation [6], and molecular dynamics and fluorescence spectroscopy measurements on model membranes of cancer cells indicate that catechins strongly interact with phospholipid bilayers [7]. It has been settled that catechins bind to the plasma membrane and interact with the lipid rafts. Catechins altered the lipid order in ht29 colon cancer cells causing inhibition of epidermal growth factor receptor [8], and affected the lipid rafts blocking activation of the c-met receptor in prostate cancer cells [9]. In addition, there is evidence that one of the principal processes whereby catechins exert their anticancer action is the induction of lipid raft mediated apoptosis [3]. Catechins may exert their effects at the membrane level or inside the cell, but in both cases, during their therapeutic route, the interaction with the membrane becomes obligatory, hence this interaction should be studied in order to get insight into their mechanism of action.

The lipid composition of the membranes is complex, and the presence of different lipid species provides particular membranes with typical features concerning thickness, permeability, and fluidity, among other properties [10]. In this respect, anionic phospholipids are relatively minor components of most biological membranes which, nevertheless, possess singular physical and biochemical properties that make them physiologically essential. The exploration of the effect of catechins on the anionic lipid component of membranes would be crucial to clear up the mechanism of action of these compounds and would also contribute to investigate other potential activities of the molecules, as it has been proposed that the partition of catechins into the lipid bilayers and their perturbing effects are related to their antioxidant and antibacterial activities [11,12], and it has been suggested that catechins may exert their effects on membrane function by a common bilayer mediated mechanism [13].

Phosphatidylserine plays extensive functional roles both inside and outside the cell, from the classic signal in the coagulation cascade [14] to the recognition of the apoptotic cells [15]. Phosphatidylserine is also the favorite target for a variety of proteins with specific motifs and domains [16]. Interestingly, it has been demonstrated that this phospholipid is overexpressed in a lot of cancer cell types, including breast cancer, glioblastoma, and astrocytoma [17], and, thus, it has been established as a cancer biomarker [18]. Recently, it has been shown that the specific interaction of two antitumor peptides with phosphatidylserine seems to regulate their specificity for cancer membranes [19], and that a small GTPase, which is commonly mutated in human cancers, displays a precise binding specificity for phosphatidylserine [20]. Given all the above, it would be important to determine the influence of catechins on phosphatidylserine membranes.

In spite of the exceptional anticancer characteristic of catechins, they present an important restraint: their poor bioavailability, which is associated to their low stability in neutral of slightly alkaline solutions and their inefficiency to conveniently traverse cellular membranes [21]. There are extensive efforts to develop new catechin-derived compounds and improve such bioavailability issue. An important catechin derivative is TMBC, which has been established to display a high antiproliferative activity against malignant melanoma cells [22,23,24].

**Figure 1:**
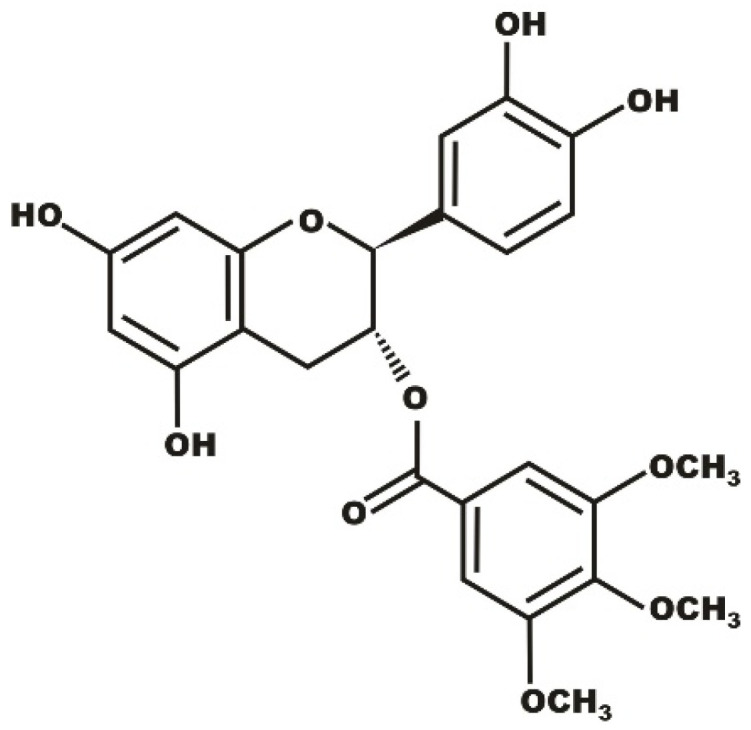
Chemical structure of TMBC.

The amphiphilic nature of TMBC (Figure 1) indicates that the membrane could be a potential site of its action, therefore, the study on the influence of this semisynthetic catechin on the lipid component of membranes is crucial in order to get insight into the mechanism of anticarcinogenic action of this compound. The examination of this TMBC-membrane interaction could also shed light on the mechanisms underlying other demonstrated beneficial effects of catechins [25]. It has been shown that TMBC is incorporated into membranes composed of the most abundant phospholipids in eukaryotic membranes phosphatidylcholine [26] and phosphatidylethanolamine [27], where it perturbed the structural properties of these phospholipid bilayers. In order to further characterize the molecular interaction between this semisynthetic catechin and the lipidic component of the membrane, we present in this work an experimental study using differential scanning calorimetry (DSC), X-ray diffraction, Fourier transform infrared (FTIR) spectroscopy, and flurescence polarization, combined with a computational molecular dynamics study of the interaction between TMBC and biomimetic model systems composed by the anionic phospholipid 1,2-dimyristoyl-sn-glycero-3-phospho-L-serine (DMPS).

## 2. Results and Discussion

The interaction between TMBC and anionic membranes was carried out by employing DMPS model bilayers. We made use of DSC in order to get information concerning the effect of TMBC on the thermotropic gel to liquid crystalline phase transition of phosphatidylserine and of small (SAXD) and wide (WAXD) angle X-ray diffraction techniques to get insight into the overall structural properties of DMPS. FTIR spectroscopy and fluorescence polarization was applied to investigate the interaction of the catechin with a different part of the phospholipid molecule, and eventually molecular dynamics simulation was used to establish the position and dynamics of TMBC inside DMPS bilayer.

### 2.1. DSC

**Figure 2:**
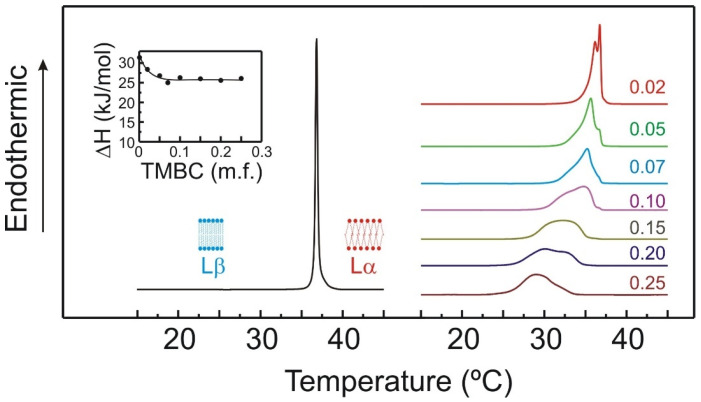
DSC heating thermograms for pure DMPS (left) and DMPS containing TMBC at different concentrations (right). The inset shows the enthalpy change for the main gel to liquid-crystalline phase transition. TMBC molar fractions are expressed on the right side of the thermograms.

The perturbation exerted by TMBC on the thermotropic phase transition of DMPS is shown in Figure 2. Upon heating, pure DMPS exhibits only one highly cooperative endotherm starting at 36.2 °C, in agreement with earlier data [28,29], which corresponds to the gel (Lβ) to liquid crystalline (Lα) phase transition. The presence of even low amounts of TMBC like 0.02 molar fraction causes the apparition of a second peak at lower temperatures, the effect of increasing concentrations of TMBC has the effect of broadening the temperature range of the transition and shifting the transition to lower temperatures. The thermograms are composed by different peaks or shoulders, which may indicate that different domains are present in the bilayer. The inset in Figure 2 shows that the presence of low proportions of TMBC produces a decrease of near 20% of the enthalpy change of the transition, and that from 0.07 molar fraction up no further decrease is found. These effects of TMBC on the gel to liquid-crystalline phase transition thermograms of phosphatidylserine membrane indicate that TMBC incorporates into the phosphatidylserine bilayers, where it is able to perturb the acyl chains of the phospholipids, reducing the cooperativity of the transition, lowering the phase transition temperature, and decreasing the enthalpy change of the transition.

### 2.2. X-ray Diffraction

**Figure 3:**
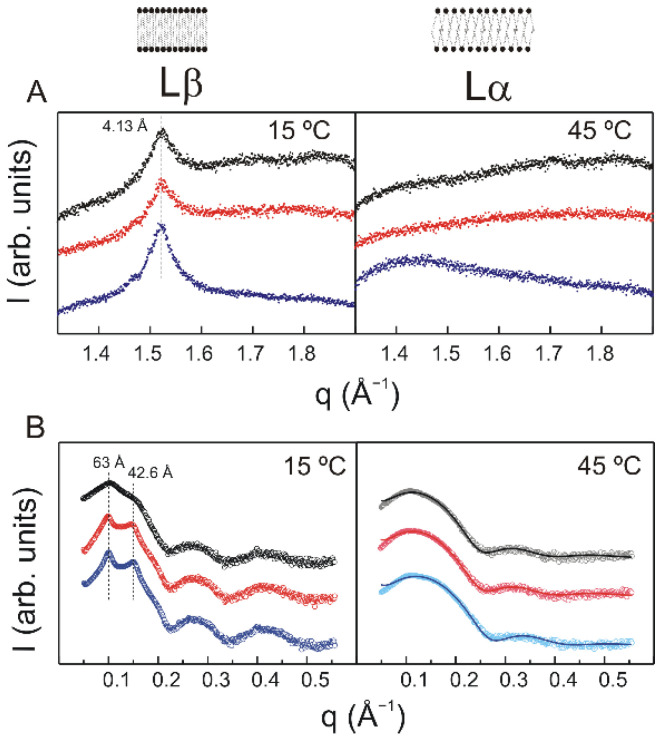
(**A**) Intensity (arbitrary units) vs. scattering vector (q) for WAXD profiles of pure DMPS (top) and DMPS containing TMBC at 0.07 (middle) and 0.20 molar fraction (bottom) at different temperatures. (**B**) Intensity (log scale in arbitrary units) vs. scattering vector (q) for SAXD profiles of pure DMPS (top) and DMPS containing TMBC at 0.07 (middle) and 0.20 molar fraction (bottom) at different temperatures. Solid lines at 45 °C represent the best fit to the experimental patterns using the GAP program.

Measurements in the WAXD region provide information about the packing of the phospholipid acyl chains. Figure 3A displays the WAXD pattern corresponding to pure DMPS and DMPS containing TMBC. At 15 °C (Figure 3A, left) pure DMPS shows a symmetric reflection centered at 4.13 Å indicative of a conventional Lβ gel phase, in which the acyl chains are packed parallel to the bilayer normal on a regular hexagonal lattice. At 45 °C (Figure 3A, right), pure DMPS displays a very broad component centered at 4.4 Å, which is typical of the disordered Lα liquid crystalline phase, these values being in consonance with previous data [30]. As shown in Figure 3A, the presence of TMBC does not alter the packing of the DMPS acyl chains neither below nor above the phase transition temperature.

Measurements in the SAXD region allows to determine the macroscopic structure itself, with the location of the larger first order reflection providing the interlamellar repeat distance in the lamellar phase, which is comprised of the bilayer thickness and the thickness of the water layer between bilayers [31]. The SAXD pattern for pure DMPS in the gel phase shown in Figure 3B (left) reveals a diffraction peak with an interlamellar repeat distance of 63 Å, this distance decreasing to 57.8 Å in the liquid crystalline phase (Figure 3B, right). These data are within the range of those of previous studies [29,32]. It is noteworthy that the SAXD pattern or pure DMPS shows a broad reflection, instead of the typical sharp Bragg diffraction peak characteristic of ordered multilamellar systems. The explanation of the latter being that the Na^+^ concentration used in our system, is unable to completely shield the negatively charged bilayer surface, and the remaining electrostatic repulsive forces prevent the orderly apposition of bilayers of this negatively charged phospholipids [33]. Furthermore, it has been described that in continuous swelling lipid-water systems at high hydration levels, multilayer stacking disorders results in a broadening of the SAXD lines [34]. A careful look to the SAXD pattern of pure DMPS in the gel phase also reveals the presence of an additional broader and weaker reflection at a lower distance. The presence of this additional weak reflection in DMPS has been reported previously [30] and is in line with the early observation that, in some cases, phosphatidylserine dispersions showed weaker diffraction lines from multilamellar structures of different dimensions in addition to the principal diffraction [35].

In the presence of TMBC, the DMPS SAXD pattern in the gel phase (Figure 3B, left) shows two clear sharp Bragg diffraction peaks. This is at difference with the broad reflection appearing in the pure phospholipid, and indicates that multilamellar vesicles have been formed. It might be suggested that the presence of TMBC somehow perturbed the interactions between the adjacent bilayer, allowing the formation of more membrane stacks. These reflections appear at distances of 63 Å and 42.6 Å. The sharp reflection at 63 Å coincides with the interlamellar distance of the DMPS gel phase, and the sharp reflection at 42.6 Å most likely corresponds to the weak reflection, which appeared at lower distances in the pure DMPS pattern. However, this reflection at lower distances cannot be indexed according to the same lattice as the 63 Å bilayer spacing. Considering that TMBC has been shown to be able to induce the formation of an interdigitated gel phase in phosphatidylcholine system [26], we suggest that this lower distance reflection originates from a partially interdigitated gel phase. TMBC induced a complete interdigitated gel phase in phosphatidylcholine bilayers [26], but in our phosphatidylserine system there is always a mixture of two phases, the common gel (Lβ) and the interdigitate gel phase (LβI). Several recent studies have established that the two leaflets of biological membranes most likely interact through interdigitation between the acyl chains of the phospholipids, and have suggested that saturated phosphatidylserines play a unique function in this process [36]. For that reason, it might be suggested that the weak reflection, which appeared at a lower distance in the diffraction pattern of pure DMPS correspond to a minor proportion of the DMPS bilayer, which in our system is organized already in a minor partially interdigitated phase. The presence of the additional low interlamellar distance precludes a global data analysis in term of a single phase, as the simple bilayer model commonly used in the global analysis routines [37] does not fit the SAXD pattern.

In the liquid crystalline phase (Figure 3B, right) the incorporation of TMBC does not perturb the bilayer structure as extremely, and the data were adequately fitted with the usual bilayer model of the global analysis program (GAP). We found close d_B_ values of 50.9 Å and 50.5 Å for the pure DMPS and in the presence of TMBC 0.7 molar fraction systems, respectively, however, we found a marked thinning of the bilayer thickness in the presence of TMBC 0.20 molar fraction (d_B_ = 48.4 Å).

In order to match the integration of the proteins into the membrane, it is critical that the phospholipid acyl chains and the neighboring hydrophobic part of the proteins interact in a particular manner [38]. When the hydrophobic width of the membrane is different from the dimension of the hydrophobic portion of the protein, a hydrophobic mismatch arises. This alteration of the membrane characteristics might be able to perturb the behavior of proteins that depend on lipid interactions, as these alterations can generate modifications in the structure of the protein [39]. Taking into account that lipid interacting proteins are implicated in critical cellular activities, the reduction of the membrane thickness accomplished by TMBC could be conclusive from the point of view of the hydrophobic mismatch and shows as probable that it might contribute to the mechanism of action of this catechin derivative.

**Figure 4:**
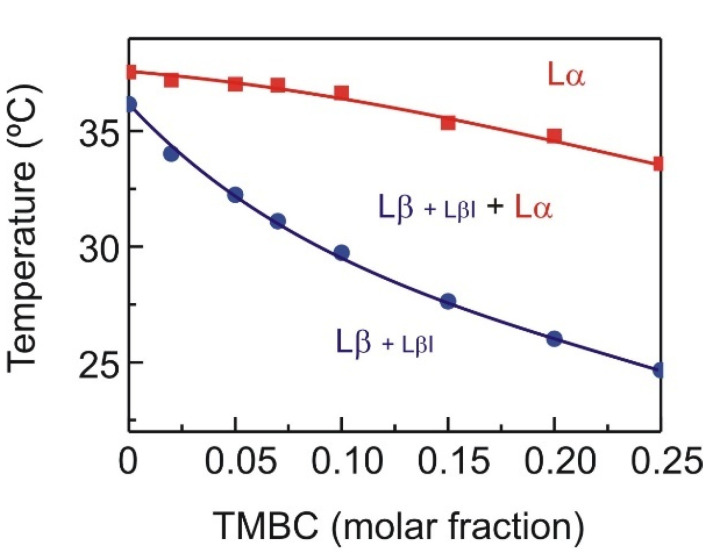
Partial phase diagrams for DMPS in DMPS/TMBC mixtures. Circles and squares were obtained from the onset and completion temperatures of the main gel to liquid crystalline phase transition, respectively. Circles, solidus line; squares, fluidus line. The phase designations are as follows: Lβ, gel phase; LβI, interdigitated gel phase; Lα, liquid crystalline phase (fluid phase).

The phase transitions temperatures obtained from DSC measurements and the structural information from the X-ray diffraction experiments have been used to construct a partial phase diagram for the DMPS component in mixtures with TMBC, and this is presented in Figure 4. Both the solidus and the fluidus lines display a near ideal behavior in the whole range of TMBC concentrations, with their temperatures decreasing as more TMBC is present into the bilayer, being the decrease of the fluidus line less apparent than the decrease of the solidus one. The system evolves from a gel phase, in which the common gel phase (Lβ) is present together with a minor population of an interdigitated gel phase (LβI), to a liquid crystalline phase (Lα) through a coexistence region that is wider, as more TMBC is present in the system. The solidus line shows good miscibility in the whole range of the TMBC concentration, which is dissimilar from the previously reported behavior of TMBC in systems composed of zwitterionic phospholipids [26,27], where stoichiometric compounds are formed at a high concentration of TMBC. The latter reveals the importance of the charge of the phospholipid head group in the interaction of this catechin with the membrane.

### 2.3. FTIR Spectroscopy and Fluorescence Polarization

It is possible to obtain valuable knowledge about the molecular interplay of the distinct parts of the phospholipid molecule from its FTIR spectrum. There are two main informative absorption regions, one is the CH_2_ stretching bands, which report on the properties of the acyl chains and the other is the C=O stretching band, which report on the interfacial region of the molecule.

**Figure 5:**
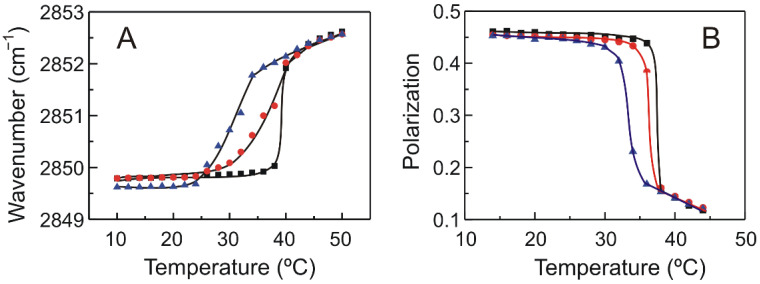
(**A**) Temperature dependence of the maximum of the symmetric methylene stretching vibration band, sν(CH_2_), exhibited by pure DMPS (squares) and DMPS containing TMBC at 0.07 (circles) and 0.20 (triangles) molar fractions. (**B**) Steady state fluorescence polarization as a function of temperature of DPH incorporated into membranes composed of pure DMPS (squares) and DMPS containing TMBC at 0.07 (circles) and 0.20 (triangles) molar fractions.

There are several absorption bands arising from the different C-H stretching vibrations, but the most helpful one is the CH_2_ symmetric band, because it is not affected by absorption from other groups. This CH_2_ symmetric band is responsive to alterations in the motion and order of the hydrocarbon chains of the phospholipid molecule [40]. When the phospholipid undergoes the gel to fluid transition, the maximum of this band shift to a higher wavelength with a concomitantly increase of the width of the band [41]. These changes are typical of the gel to fluid phase transition of hydrated phospholipids. The variation of the maximum of the CH_2_ symmetric vibration as the temperature is increased is presented in Figure 5A, showing systems composed of pure DMPS and systems with mixtures of DMPS and TMBC. As has been previously reported [28], at temperatures below the phase transition, the maximum of the band is located around 2849.5 cm^−1^, and at temperatures above the phase transition the maximum is shifted to higher wavelengths around 2852.5 cm^−1^. When the phase transition takes place, the presence of a high proportion of gauche conformers giving rise to a more disorder acyl chains is responsible for the shift of the maximum to higher wavelengths [42]. As seen in Figure 5A, the incorporation of TMBC into DMPS bilayers determines that the temperature of the transition appears at lower values, this is in accordance with the shift of the phase transition temperature to lower values determined by DSC (Figure 2). However, the presence of TMBC did not alter the maximum wavelength of the band neither at temperatures below nor above the phase transition, which suggest that TMBC did not modify the fluidity of the bilayer either in the gel phase or the fluid phase.

Fluorescence polarization measurements, using a 1,6-diphenyl-1,3,5-hexatriene (DPH) probe, were carried out to establish this absence of changes in membrane fluidity. Figure 5B shows the fluorescence polarization of DPH incorporated into pure DMPS systems and those containing TMBC, as a function of temperature. The sharp drop in polarization values detected in the pure DMPS sample reflects the increase in membrane fluidity taking place during the gel to liquid-crystalline phase transition. The presence of TMBC caused a broadening and shifting of the transition to lower temperatures, in line with the evidence shown above. Interestingly, at temperatures both below and above the phase transition, the presence of TMBC does not affect the polarization values of the probe, indicating that no change in fluidity is taken place. The latter is in agreement with the lack of effect on the methylene region exerted by TMBC at these temperatures, as shown in Figure 5A.

**Figure 6:**
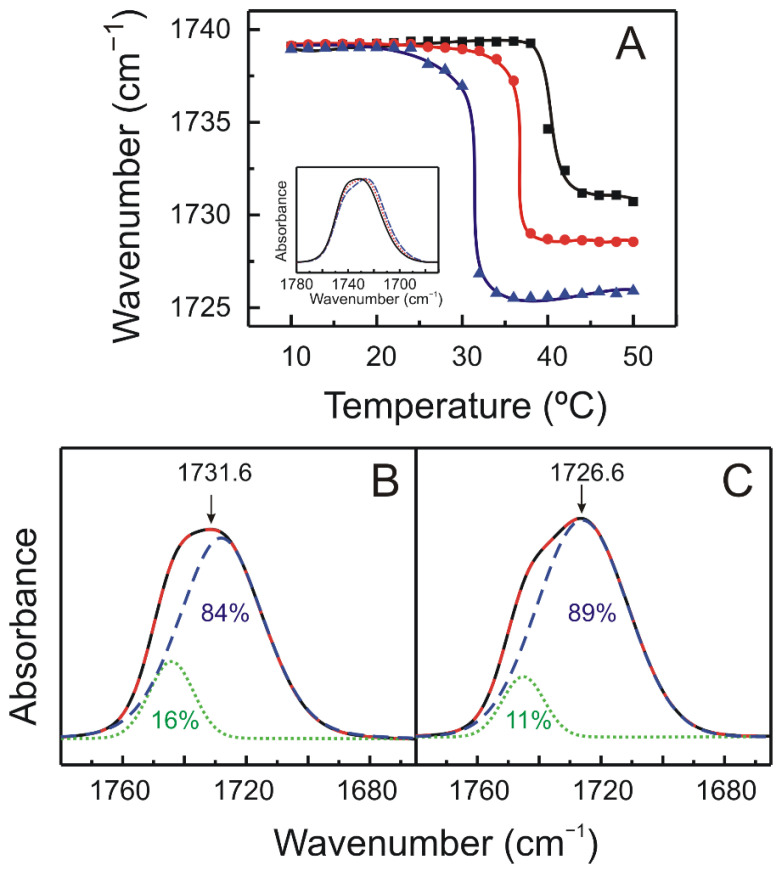
(**A**) Temperature dependence of the maximum of the ester carbonyl stretching band, ν(C=O), exhibited by pure DMPS (squares) and DMPS containing TMBC at 0.07 (circles) and 0.20 (triangles) molar fractions. The inset shows the 1780–1670 cm^−1^ spectral region containing the absorption band originating from the ester carbonyl stretching band of pure DMPS (solid line) and DMPS containing TMBC at 0.07 (dotted line) and 0.20 (dashed line) molar fractions, at 45 °C. FTIR spectra illustrating the components of the ester carbonyl stretching band, exhibited by (**B**) pure DMPS and (**C**) DMPS containing TMBC at 0.20 molar fraction at 45 °C. The different lines represent: solid line, observed baseline corrected spectra; large dashed line, fitted spectra; dotted line, estimates of the component band appearing at 1742 cm^−1^; and short dashed line, estimates of the component band appearing at 1728 cm^−1^, as determined by the curve fitting. The arrows point to the maximum of the ester carbonyl stretching band.

The thermotropic phase change displayed by anionic phospholipids is followed by very apparent changes in the contours of the ester carbonyl stretching band, ν(C=O). The features of this absorption band are sensitive to the conformation, hydration state, and the degree and nature of hydrogen-bonding interactions in the polar/apolar interfaces of phospholipids bilayers [40]. As shown in the inset of Figure 6A, the carbonyl stretching band of DMPS is a fairly broad band around 1760–1690 cm^−1^, and it is known that the carbonyl groups of diacylphospholipids may be found in lipid vesicles in hydrogen bonded and non-hydrogen bonded states, their proportions depending on the physical state of the phospholipid bilayer [40]. Pure DMPS ester carbonyl stretching band is considered to be a summation of two component bands centered near 1742 cm^−1^ and 1728 cm^−1^ [28,29]. The relative intensities of these component bands reflect the contribution of subpopulation of non-hydrogen bonded and hydrogen bonded carbonyl groups [43]. Figure 6A shows the temperature dependence of the frequency of the absorbance maximum of the carbonyl stretching band of the FTIR spectra corresponding to pure DMPS and DMPS/TMBC systems. For pure DMPS, the gel to liquid crystalline phase transition produced a shift of the maximum frequency to lower wavenumbers, in accordance with the increase in intensity of the underlying component band at 1728 cm^−1^, attributed to a higher amount of hydrogen bonded carbonyl groups resulting from a phase state-induced increase in the hydration of the polar–apolar interface. In accordance with the methylene stretching band results commented above (Figure 5A), the shift of the phase transition to lower temperatures produced by the presence of TMBC can also be noticed following the maximum of the carbonyl band illustrated in Figure 6A. It is interesting to note that, in the liquid crystalline phase, the presence of increasing concentrations of TMBC caused a shift of the maximum of the carbonyl band to lower frequencies as compared with the pure phospholipid. This decrease in frequency, which can be also clearly observed in the inset of Figure 6A, suggests an increase in the proportion of the hydrogen bonded carbonyl groups, implying that TMBC interacts with the interfacial region of the anionic phospholipid bilayer, increasing the hydrogen bonding pattern of the phospholipid.

We performed a simulation by a Gaussian–Lorentzian function in order to fit the different C=O stretching spectra and obtain information on the hydrogen bond pattern in the absence and the presence of TMBC. The C=O spectra of DMPS was best fitted to two components located near 1742 cm^−1^ and 1728 cm^−1^ (Figure 6B), the proportion of the non-hydrogen bonded C=O being near 16%, which is in accordance with earlier reports [28]. The proportion of non-hydrogen bonded C=O diminished to 11% when TMBC was incorporated at a 0.07 molar fraction and to 11% when the molar fraction was 0.20 (Figure 6C). Simultaneously with the latter, the presence of TMBC produces an increase of the proportion of hydrogen bonded C=O, evidencing that TMBC alters the hydrogen bonding pattern of the phospholipid.

### 2.4. Molecular Dynamics

The area per lipid was calculated as the area of the x y plane of the simulation box divided by the number of lipids in each leaflet. The area per lipid of pure DMPS bilayers in the liquid crystalline phase was 56 Å^2^, this value being among the reported data [44,45], however, an increment in the area per lipid was observed in the presence of TMBC (61 Å^2^). The membrane thickness was computed calculating the phosphorous atoms distance among both leaflets. The bilayer thickness of pure DMPS in the liquid crystalline phase was 38.8 Å. This value decreases to 35.4 Å in the presence of TMBC, which agrees with the thinning effect observed in the SAXD experiments (Figure 3B).

The *gauche* to *trans* ratio conformations of the lipid acyl chains was calculated to examine the possible changes in the hydrocarbon core of the bilayer by the presence of TMBC. A value of 0.4 was determined for the *gauche* to *trans* ratio in pure DMPS, and no changes were observed in the presence of TMBC, which is in agreement with the FTIR on the methylene absorption band and the fluorescence polarization experiments (Figure 5) commented above.

The number of hydrogen bonds per lipid molecule between lipid carbonyl groups and water and TMBC molecules were measured. The results showed that the total number of hydrogen bonds per lipid increases from 1.34 for pure DMS to 1.48 for the system in the presence of TMBC. This increase is due to the new hydrogen bonds established between the carbonyl groups of the phospholipid and the hydroxyl groups of TMBC, and it is in agreement with the increase in the number of hydrogen bonds determined by the shift of the maximum of the carbonyl absorption band to lower wavelengths obtained by FTIR and the contribution of the two bands component (Figure 6).

It is known that in the case of phosphatidylserine systems, neighboring lipids could be bound by hydrogen bonds between amino groups and phosphate groups of the phospholipids. It is conceivable that the incorporation of TMBC into the phosphatidylserine bilayer should perturb this bonding pattern. We evaluate the number of hydrogen bonds between different DMPS molecules and found a value of 0.70 hydrogen bonds per phospholipid molecule; this value decreased to 0.64 in the presence of TMBC. The latter support the idea that the incorporation of TMBC into the bilayer, in addition to be able to perturb the hydrogen bonding pattern of the carbonyl group with water, is also able to alter the intermolecular hydrogen bonding of the phosphatidylserine bilayer.

It is interesting to note that we have shown that the presence of TMCG in the liquid crystalline phase produced a marked thinning of the bilayer, which might be explained by induced partial interdigitation. In accordance with the relation between the formation of the interdigitated phase and its association with an increase of the distance between the lipid heads in the membrane plane, we have indeed shown above that the area per lipid increased in the presence of TMCG. All the above support the hypothesis of formation of a partial interdigitated phase.

**Figure 7:**
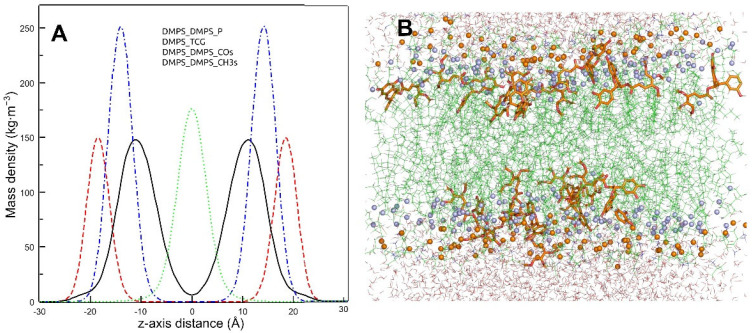
(**A**) Mass density profiles along the z-axis of the simulation box of DMPS–TMBC membrane at 323 K. TMBC molecules in solid line, phosphorus atoms in dashed line, lipid carbonyl groups in dash-dot line, and lipid terminals methyl carbon atoms in dotted line. (**B**) Final snapshot of the simulation box of the DMPS–TMBC membranes. Water molecules are shown in lines, TMBC in sticks, DMPS in lines, lipid carbonyl groups in light spheres, and phosphorous atoms in dark spheres.

The mass density profiles of the simulated membrane in the presence of TMBC is shown in Figure 7A. The lipid phosphorous atoms were included to label the polar head region, the lipid terminal methyl groups to label the center of the membrane, and the lipid carbonyl groups to label the position of hydrogen bonds. In the DMPS membrane, TMBC molecules were mainly located in the center of the lipid hydrocarbon chains between the phosphorous atoms and the terminal methyl groups, overlapping with the carbonyl groups of the phospholipid, enabling the formation of hydrogen bonds, and with essentially no molecules located in the center of the membrane. The representative snapshot of the DMPS–TMBC membrane (Figure 7B) shows that TMBC molecules are present as monomers and form small aggregates closer to the polar head group of DMPS. This location is different from that of TMBC in phosphatidylcholine bilayers, where the catechin extended more toward the center of the membrane [26], and from TMBC in phosphatidylethanolamine bilayers [27], where the catechin formed large aggregates, which reached the center of the bilayer. TMBC appears to locate differently in specific phospholipid bilayers, showing convincingly that the characteristic of the phospholipid head group plays a central role in the interaction of the catechin with the membrane.

**Figure 8:**
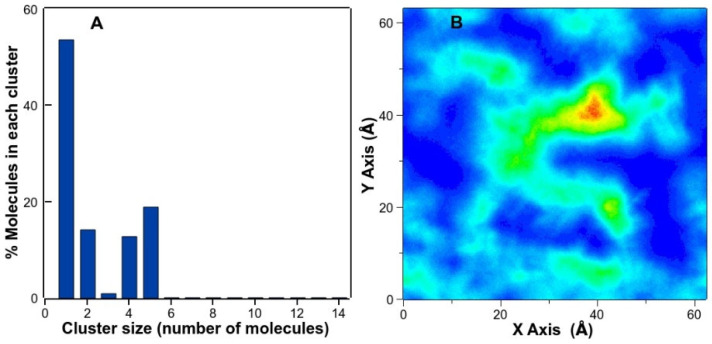
(**A**) Cluster size distribution of TMBC molecules in the DMPS membrane. (**B**) Density map of TMBC molecules of the DMPS/TMBC membrane corresponding to the x y plane of the simulation box. TMBC density increases from blue to red color.

The proclivity of TMBC to form aggregates was examined by determining the cluster size distribution of TMBC in the bilayer, and it was calculated as the number of TMBC molecules that are found in the analyzed trajectory within a distance of 3 Å. As can be observed in Figure 8A, approximately half of the TMBC molecules are present as monomer, while the other half are found forming small aggregates of 2–5 molecules. Figure 8B shows the density map of TMBC in the x y plane of the bilayer displaying the formation of different clusters along the plane of the bilayer. The presence of these different clusters in the liquid crystalline bilayer may explain the presence of different domains, as suggested in the DSC thermograms, and the absence of effect on the enthalpy change of the transition when TMBC is present at high concentration (Figure 2).

## 3. Materials and Methods

### 3.1. Materials

DMPS (sodium salt, >99% TLC) was obtained from Avanti Polar Lipids Inc. (Birmingham, AL). Phospholipid concentration was determined by phosphorous analysis [46]. (–)-Catechin, 3,4,5-trimethoxybenzoyl chloride, and DPH were obtained from Sigma Chemical Co. (Madrid, Spain). Synthesis of TMBC started from commercially available catechin, and the reaction sequence was designed to avoid problems associated with unspecific blockage of the 3-hydroxy group of catechin following the benzylation reaction with benzyl bromide and potassium carbonate. The compound was esterified with previously prepared 3,4,5-trimethoxybenzoyl chloride in a dichloromethane solution in the presence of 4-dimethylaminopyridine. Finally, the benzyl groups were removed by hydrogenolysis to produce TMBC in high yield and purity (>98%). Data characterization was done by means of ^1^H an ^13^C NMR, heteronuclear multiple quantum coherence, and electrospray ionization mass spectrometry. Detailed data on experimental procedure for synthesis, purity, and characterization, have been previously published [22]. Purified water was deionized in a Milli-Q equipment (Merck Millipore, Bedford, MA, USA), and filtered through 0.24 μm filters prior to use. All other reagents were of the highest purity available.

### 3.2. DSC

The lipid mixtures for DSC measurements were prepared by combination of chloroform: methanol (4:1) solution containing DMPS and the appropriate amount of TMBC (in ethanol), as indicated. The organic solvent was evaporated under a stream of dry N_2_, and the last traces of solvent was removed by further 3 h evaporation under high vacuum. To the dry samples, 0.5 mL of a buffer containing 150 mM NaCl, 0.1 mM EDTA, 10 mM Hepes, pH 7.4 was added, and multilamellar vesicles were formed by vortexing the mixture, at a temperature above the gel to liquid-crystalline phase transition temperature of the phospholipid. Experiments were performed using a MicroCal DSC PEAK calorimeter (Malvern Panalytical, Malvern, UK). The final phospholipid concentration was 1.5 mM, and the heating scan rate was 60 °C h^−1^. The integral of the heat capacity over temperature gives the calorimetric enthalpy change for the transition, and accordingly, peak areas under the thermograms relative to the baseline were determined as a direct measurement of the enthalpy change. Data were analyzed using ORIGIN v7.0383 (Northampton, MA, USA) software provided by MicroCal. The construction of partial phase diagrams was based on the heating thermograms for a given mixture of phospholipid and TMBC at various TMBC concentrations. The onset and completion temperatures for each transition peak were obtained from the heating thermograms taken at the points of intersection of the tangents to the leading edges of the endotherms and the baselines and were plotted as a function of the molar fraction of TMBC. These onset and completion temperatures points formed the basis for defining the boundary lines of the partial temperature-composition phase diagram.

### 3.3. X-ray Diffraction

Simultaneous SAXD and WAXD measurements were carried out using a modified Kratky compact camera (MBraum-Graz-Optical Systems, Graz, Austria) which employs two coupled linear position sensitive detectors (PSD, MBraum, Garching, Germany). Nickel-filtered Cu Kα X-rays were generated by a Philips PW3830 X-ray Generator operating at 50 kV and 30 mA. The q (scattering vector) range covered (q = 4π sin θ/λ; where 2θ is the scattering angle and λ = 1.54 Å the selected X-ray wavelength) was between 0.05 and 0.6 Å^−1^ for SAXD and from 1.32 to 1.95 Å^−1^ for WAXD, respectively. Samples for X-ray diffraction were prepared by mixing 10 μmol of DMPS and the appropriate amount of TMBC in organic solvents, and multilamellar vesicles were formed as described above. After 5 min centrifugation at 12,000 rpm, the pellets were placed in a steel holder, which provided good thermal contact to the Peltier heating unit, with cellophane windows. Exposure times were 10 min, allowing 10 min prior to the measurement for temperature equilibration. Background corrected SAXD data were analyzed using the program GAP obtained from the author [37,47]. This program allowed to retrieve the membrane thickness, d_B_ = 2 (Z_H_ + 2σ_H_) from a full q-range analysis of the SAXD patterns [48]. The parameters Z_H_ and σ_H_ are the position and width, respectively, of the Gaussian used to describe the electron-dense headgroup regions within the electron density model.

### 3.4. FTIR Spectroscopy

Samples for the infrared measurements containing 10 μmol of DMPS and the appropriate amount of TMBC were formed in 75 μL of the same buffer prepared in D_2_O as described above. Samples were placed in between two CaF_2_ windows (25 × 2 mm) separated by 25 mm Teflon spacers (Thermo Fisher Scientific, Madison, WI, USA) and transferred to a Symta cell mount (Symta, Madrid, Spain). Infrared spectra were acquired in a Nicolet 6700 FTIR spectrometer (Thermo Fisher Scientific, Madison, WI, USA). Each spectrum was obtained by collecting 256 interferograms with a nominal resolution of 2 cm^−1^. The equipment was continuously purged with dry air in order to minimize the contribution peaks of atmospheric water vapor. The sample holder was thermostatized using a Peltier device (Proteus system from Nicolet). Spectra were collected at 2 °C intervals, allowing 5 min equilibration between temperatures. The D_2_O buffer spectra taken at the same temperatures were subtracted interactively using either Omnic (Thermo Electron Corporation, Walthman, MA, USA) or Grams (Galactic Industries, Salem, NH, USA) software.

### 3.5. Steady-State Fluorescence Polarization

Samples for fluorescence polarization were prepared by a combination of chloroform: methanol (4:1) solution containing DMPS, the appropriate amount of TMBC (in ethanol) as indicated, and DPH in chloroform at a probe:phospholipid molar ratio of 1:500. The organic solvents were evaporated, and the last traces of solvents were removed by further evaporation under high vacuum. From the dry samples, multilamellar vesicles were formed as described above. To ensure that depolarization due to light scattering was not occurring, the value of polarization was measured before and after diluting the sample. In cases where dilution gave an increase in polarization, the samples were diluted until the value had reached a maximum and was no longer concentration dependent. The optical density of the samples at the excitation wavelength was 0.036, and TMBC demonstrated no absorbance at the excitation and emission wavelengths of DPH and therefore the inner filter effect was negligible. Steady-state fluorescence polarization measurements were performed with a PTI Quantamaster spectrofluorometer (Photon Technology, Birmingham, NJ, USA) equipped with motorized polarizers. Quartz cuvettes with a path length of 10 mm were used. The cell holder was thermostatized using a Peltier device, and the measurements were taken under continuous stirring. For monitoring DPH fluorescence, the excitation wavelength was set at 358 nm, and emission was monitored at 430 nm. The sample temperature was allowed to equilibrate for 5 min before fluorescence was recorded during a 60 s interval. The excitation shutter was kept closed during heating to the next temperature, in order to minimize any photoisomerization of DPH. Steady-state fluorescence polarization values were calculated from the following Equation (1):

P = (I_VV_ − G I_VH_) / (I_VV_ + G I_VH_), (1)

where I_VV_ and I_VH_ are the fluorescence intensities with the excitation polarizer oriented vertically and the emission polarizer oriented vertically and horizontally, respectively. G is the grating factor, calculated as the ratio of the efficiencies of the detection system for vertically and horizontally polarized light, and is equal to I_HV_/I_HH_.

### 3.6. Molecular Dynamics

The 3D molecular structure of TMBC was constructed from (−) Catechin gallate chemical structure obtained from the PubChem Substance and Compound database [49] through the unique chemical structure identifier CID 6419835. All MD simulations were done using GROMACS 5.0.7 and 2018.1 [50] in the Computational Service of the University of Murcia (Spain). CHARMM36 force field parameters for DMPS, TMBC, water, Cl^−^ and Na^+^ were obtained from CHARMM-GUI [51,52,53]. The membrane bilayers were formed by 2 leaflets oriented normal to the z-axis with a total of 128 molecules of DMPS with and without 14 molecules of TMBC, and a water layer containing a total of 6400 water molecules (TIP3 model), 143 sodium ions, and 15 chloride ions. The initial membrane structures were built with the aid of Packmol software (University of Campinas, Campinas, Brazil) [54].

Systems were simulated using the NpT-ensamble at 323 K. Pressure was controlled semi-isotropically at a pressure of 1 bar and compressibility of 4.5 × 10^−5^ bar^−1^. The cutoffs for van der Waals and short-range electrostatic interactions were 1.2 nm, and a force switch function was applied between 1.0 and 1.2 nm [55]. Simulations were initiated by a 20 ns run, using the V-rescale thermostat and the Berendsen barostat [56], followed by a 180 ns run using the Nose–Hoover thermostat [57] and the Parrinello–Rahman barostat [58]. Graphical representations were done with PyMOL 2.3.0 (Schrödinger, New York, NY, USA) [59]. Analysis of the trajectories were done over the last 60 ns using the Gromacs tools.

## 4. Conclusions

The objective of this work was to characterize the molecular interactions of the anticarcinogenic semisynthetic catechin derivative TMBC with anionic phospholipids membranes formed by DMPS. Our DSC data supported that TMBC is able to incorporate into DMPS bilayers and to embed between the molecules of phospholipids, where it can reduce the cooperativity and lower the transition temperature of the gel to liquid-crystalline phase transition. The presence of multiple peaks in the thermograms suggested that different TMBC-phospholipids domains were formed. X-ray diffraction measurements indicated that TMBC was able to partially induce the formation of interdigitated gel phase in DMPS, and in the liquid crystalline phase, the presence of TMBC produced a decrease of the bilayer thickness. Infrared experiments revealed that, in the liquid crystalline phase, TMBC increased the hydrogen bonding of the carbonyl interfacial group of the phospholipid. Our molecular dynamic simulation studies were in agreement with our experimental results and showed that TMBC is located into the phospholipid palisade of DMPS, with a tendency to form small clusters. The position of TMBC in the bilayer allowed the formation of new hydrogen bonds between the carbonyl groups of the phospholipid and the hydroxyl groups of the catechin, and also granted the catechin to disturb the intermolecular hydrogen bonding between neighboring DMPS molecules. Taken together, all these results point to a strong interaction between TMBC and anionic bilayers generating physical perturbations, which could modify membrane function. We believe that these findings may contribute to the understanding of the mechanism of the anticarcinogenic action of TMBC and also of the increasingly expanding membrane related biological actions of catechins.

## Data Availability

Not applicable.
